# Automated Measurement and Control of Germination Paper Water Content

**DOI:** 10.3390/s19102232

**Published:** 2019-05-14

**Authors:** Lina Owino, Marvin Hilkens, Friederike Kögler, Dirk Söffker

**Affiliations:** Chair of Dynamics and Control, University of Duisburg-Essen, Lotharstraße 1, 47057 Duisburg, Germany; marvin.hilkens@de.bertrandt.com (M.H.); friederike.koegler@uni-due.de (F.K.); dirk.soeffker@uni-due.de (D.S.)

**Keywords:** moisture measurement, Kalman filter, model predictive control, germination paper

## Abstract

Germination paper (GP) is used as a growth substrate in plant development studies. Current studies bear two limitations: (1) The actual GP water content and variations in GP water content are neglected. (2) Existing irrigation methods either maintain the GP water content at fully sufficient or at a constant deficit. Variation of the intensity of water deficit over time for plants grown on GP is not directly achievable using these methods. In this contribution, a new measurement and control approach was presented. As a first step, a more precise measurement of water content was realized by employing the discharging process of capacitors to determine the electrical resistance of GP, which is related to the water content. A Kalman filter using an evapotranspiration model in combination with experimental data was used to refine the measurements, serving as the input for a model predictive controller (MPC). The MPC was used to improve the dynamics of the irrigation amount to more precisely achieve the required water content for regulated water uptake in plant studies. This is important in studies involving deficit irrigation. The novel method described was capable of increasing the accuracy of GP water content control. As a first step, the measurement system achieved an improved accuracy of 0.22 g/g. The application of a MPC for water content control based on the improved measurement results in an overall control accuracy was 0.09 g/g. This method offers a new approach, allowing the use of GP for studies with varying water content. This addressed the limitations of existing plant growth studies and allowed the prospection of dependencies between dynamic water deficit and plant development using GP as a growth substrate for research studies.

## 1. Introduction

Germination paper (GP) is frequently used as substrate in research concerning plants, mainly in studies involving root phenotyping [1,2], root development in early developmental stages [3], and germination [4,5]. The plants are grown on vertically arranged sheets of GP with the roots growing on the GP surface [1,2,3]. This allows image acquisition of the root system using flatbed-scanners, and therefore can be implemented in an automated, high-throughput setting as shown in Adu et al. [1]. Irrigation of the GP is applied by a pouch-and-wick system, with the lower edge of the GP immersed in a nutrient solution.

A platform for high-throughput, high resolution root phenotyping is described in Adu et al. [1], as applied to different genotypes of Brassica Rapa. Here the pouch-and-wick system is used with the lower 10 cm section immersed in nutrient solution. It is stated that the plants “showed no symptoms of mineral deficiencies when provided with an appropriate nutrient solution through the wick.”

A root phenotyping platform applied in the investigation of root growth dynamics of corn (*Zea mays* L.) is presented in Hund et al. [2]. Here the pouch-and-wick system is used with the lower 2 cm of GP immersed in nutrient solution. The root systems growing on the GP surface were scanned daily. The results showed a linear growth rate of axial roots and an exponential growth rate of lateral roots.

In an examination of the effect of phosphorus availability on root gravitropism for five different genotypes of common bean (*Phaseolus vulgaris* L.), Liao et al. [3] carried out experiments with different substrates (GP, sand, and soil) to “validate existing observations of seedlings in growth pouches with studies of older plants in soil and solid media.” The GP was also used in a pouch-and-wick configuration with the lower edge of GP hanging into nutrient solutions with different phosphorous concentrations. The results showed that root gravitropism depends on phosphorous availability and show different characteristics among genotypes. Additionally, the adaption of genotypes to phosphorous deficit is related to their ability to develop roots in the top layers of substrate.

The existing studies demonstrate the applicability of GP in studies related to root growth and development, typically under fully watered conditions. The previous studies state that the available water content is responsible for several effects, and should therefore be controlled if related research questions are to be addressed.

### 1.1. Induction of Water Deficit in GP

Exploration of water deficit effects on root growth and development with GP as a growth substrate requires the induction of water deficit in GP. Solutions of polyethylene glycol (PEG) as described in [6] have been applied in various studies to induce a water deficit in plants [4,5]. The solutions show different osmotic potentials dependent upon PEG concentration. Plants irrigated with PEG6000-solutions can therefore obtain only a part of the water contained in the solution. The osmotic potential can be measured by thermocouple psychrometry or vapor pressure osmometry [6] and is specified as pressure (measured in bar).

The effect of water deficit on sixteen different wheat genotypes in early developmental stages is described in Rauf et al. [4]. The seeds were placed between two layers of GP moistened with PEG6000-solutions of different concentration to induce water stress of different intensities. It was shown that the tested genotypes differ significantly in water stress tolerance.

In an investigation on the effect of water stress on germination indices, soluble sugar, and proline for five different genotypes of wheat, Qayyum et al. [5] employed petri dishes containing GP moistened with PEG6000-solutions at different concentrations as a growth substrate. The results show that the germination percentage, mean germination time, and coleoptile length decreased with rising water stress while soluble sugar and proline increased.

For studies involving fixed water deficit levels, PEG provides a practical solution. Where dynamic variation of water deficit levels in the course of the growth cycle is necessary, alternative approaches are needed to achieve the required water uptake.

### 1.2. Current Limitations of GP Use as a Growth Substrate

**Figure 1:**
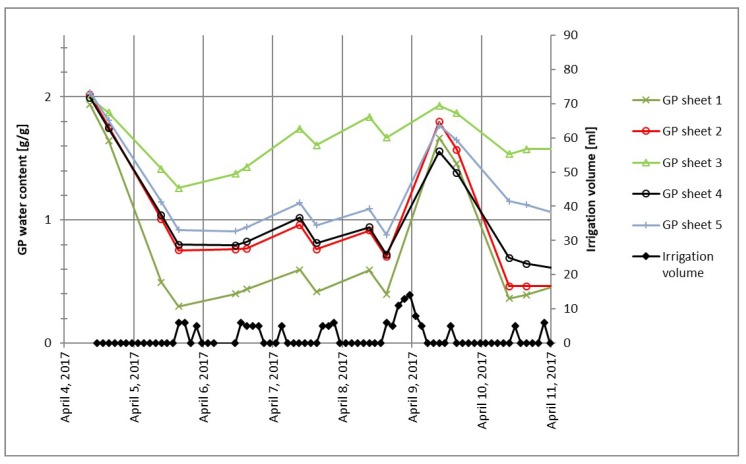
An example of germination paper (GP) water content behavior in sheets with an identical irrigation treatment.

In some studies using a pouch-and-wick system for irrigation [1,2,3], a sufficient amount of nutrient solution (not in deficit) is applied to the plants. However, the actual volumes of nutrient solution contained in the GP at different points in time as well as variations of GP water content between different GP sheets are not considered. It was observed in preliminary experiments conducted for this work that GP water content of different GP sheets strongly varied even if irrigated with the same volumes of nutrient solution at the same time, as illustrated in Figure 1. Taking into account the actual water content of each GP used in a study would result in a more accurate evaluation of the effects of plant water uptake in irrigation studies using GP as a growth substrate.

The pouch-and-wick configuration using a constant supply of nutrient solution leads to a maximal distribution of GP water content. This is defined by the equilibrium between absorption of nutrient solution and evapotranspiration. For research tasks concerning the effect of water deficit on root growth and development, improved approaches allowing for supply of water to GP below maximum level would be helpful.

In studies prospecting water stress [4,5], defined water deficit intensities are applied to GP sheets using a fixed concentration of PEG6000. This method maintains a constant water deficit level throughout and is therefore not applicable to studies requiring an application of a sequence of different water deficit intensities to the same GP sheet.

### 1.3. Resulting Targets for this Research

The contributions of this work are:

(i) The development of a method for an automated measurement of GP water content, which considers the current GP water content and variations of GP water content (between different sheets of GP and over time). Determination of the actual GP water content over time would improve the evaluation of dependencies between water uptake and plant growth and development;
(ii) The development of GP water content control approach using automated measurements and an automated irrigation system. This allows for the application of water deficit with dynamically varying intensities, expanding the scope of applicability of GP, especially for dynamic water deficit studies.

## 2. Materials and Methods

### 2.1. Ambient Conditions and Test Rig

**Figure 2:**
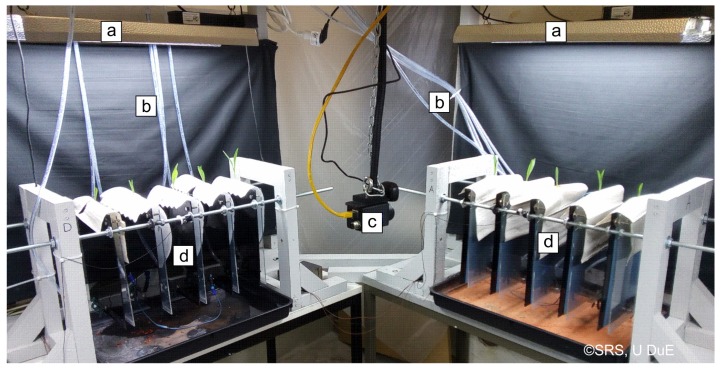
Partial view of the test rig. Two out of four test groups are shown. (**a**) Artificial illumination; (**b**) Irrigation tubes; (**c**) Infrared camera (not relevant in this work); (**d**) Test group of five specimens each consisting of a Perspex frame, GP, and a corn plant.

A system for measurement and control of GP water content was installed in a test rig for the research of plant dynamics under water stress at the Chair of Dynamics and Control, U Duisburg-Essen, Germany. A partial view of the test rig is shown in Figure 2. The test rig contains up to 20 maize plants (*Zea mays* L., Ronaldinio variety from KWS. divided into four groups of five plants each. Sheets of GP (210 × 297 mm, Hahnemühle, grade 3644, approx. 45 g) were chosen as substrate to allow a scan of the root systems. One plant was placed on each sheet. Each GP sheet was mounted on a sheet of Perspex, which was covered with a second, transparent sheet to allow scanning. Small tanks were put on the bottom right edge of the GP for irrigation, clamped between the two Perspex sheets. Approximately 3 cm of the GP was submerged in the tanks. These tanks could automatically be filled at discrete times with 5–30 mL of nutrient solution via two peristaltic pumps and a distribution system. The system for GP irrigation was described by Sattler [7].

Test runs showed that the GP could absorb 30 ml of nutrient solution from the tank in less than 2 h (=sampling time of the measurements), as long as its water content was beneath 0.67 g/g. The maximal water content of GP sheets was between 1.78 and 2.00 g/g.

Preliminary tests to determine the distribution of nutrient solution within the GP were conducted. Four pieces of GP were each marked into eight equal sections measuring 105 mm × 74 mm and submerged in nutrient solution to saturation. In experiments, the GP sheets were dried out until the mass of retained water within the GP was 50, 40, 35, and 20 g respectively, with periodic measurements taken using a precision balance to determine mass of water within the GP. As soon as each GP attained the desired mass, it was divided into the eight pre-marked sections, and water content for each individual section was determined gravimetrically.

Environmental conditions within the test rig were controlled to maintain air temperature of 21–23 ${}^{\circ }$C and artificial illumination for 14 h of simulated daylight. Illumination was provided by four 150 W, 9500 K fluorescent bulbs.

Relative air humidity was not controlled, but was measured twice a day. Relative humidity values ranged between 21–33% during the experiment.

### 2.2. GP Water Content Measurement

In pre-studies, different methods were evaluated for the measurement of GP water content (Table 1). Methods involving the direct measurement of electrical resistance were eliminated due to inconsistency in values obtained. This was taken to be due to the extremely high resistances exhibited by GP. Indirect measurement of the GP electrical resistance via the discharging of capacitors as described in Hering [8] was applied in the development of the moisture measurement system described in this work.

The relationship between the capacitor charge and electrical resistance can be described as:

(1) $$Q\left(t\right)=Q_{0}^{-\frac{t}{RC}},$$

with,

Q(t): Current capacitor charge;
    $Q_{0}$: Initial capacitor charge;
       *t*: Elapsed time;
       *R*: Electrical resistance, and;
       *C*: Capacitance.

From this relationship, the electrical resistance of the GP is obtained as:

(2) $$R=\frac{t}{C\ln\left(\frac{Q_{0}}{Q(t)}\right)}.$$

**Figure 3:**
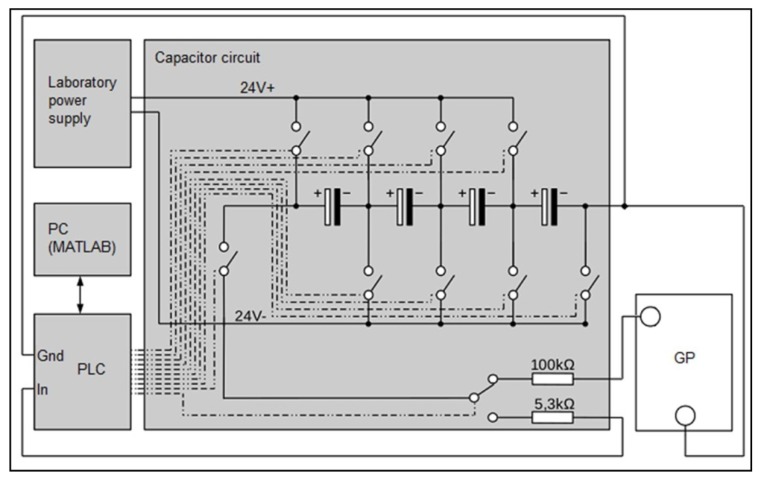
Circuit for the measurement of GP water content.

The system for the measurement of GP water content is described in Zimmermann [9]. An electric circuit as described in Hering [8] was implemented. Four capacitors (6300 μF ± 20% each) were connected in a series to facilitate a higher operating voltage as well as to increase the range of measurable resistance. The capacitors were connected to multiple relays, two resistors (100 kΩ± 5%, 5.3 kΩ± 10%), a laboratory power supply, and a programmable logic controller (PLC) (ifm CR0403). In Figure 3, the circuit for one sheet of GP is exemplarily shown. For simplification, the relays are drawn as switches connected to the PLC. Connections are denoted by chain-dotted lines. The measurement is realized using the PLC, which is connected to a PC via CAN-BUS. Every measurement is saved on the PC with a time stamp.

The measurements were implemented as follows: Each capacitor was consecutively charged to its maximum. The series-wound capacitors were connected to the GP at defined positions, starting the discharging process. The discharging process was terminated after 3 min. The remaining capacitor charge was measured via an analog current measurement via the PLC with an accuracy of ±0.2 mA. To facilitate the analog-to-digital conversion of the signal by the PLC, a sampling frequency of 10 Hz was selected.

**Figure 4:**
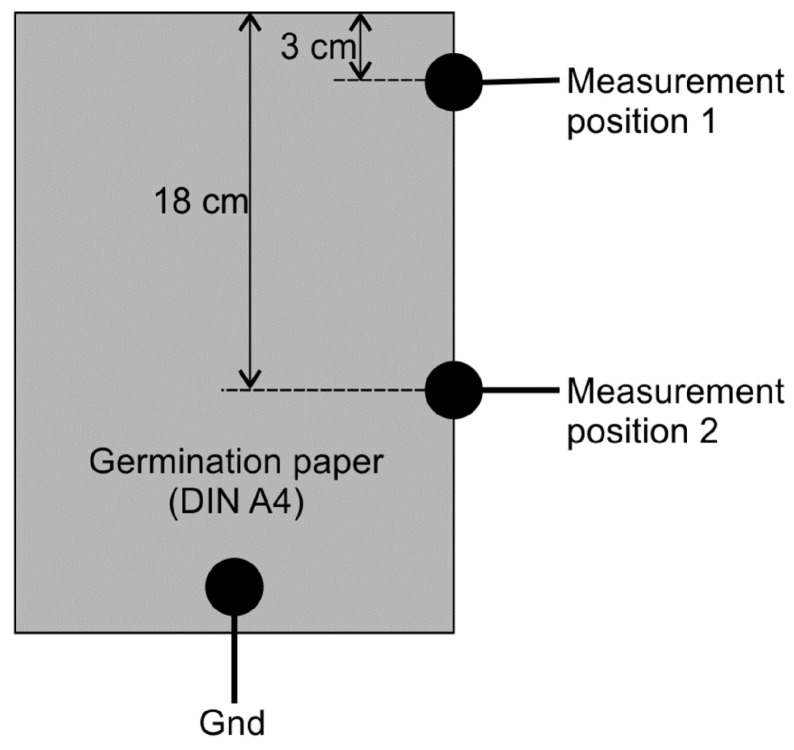
Positions of contacts for measurement of GP water content.

The water content of 8 out of 20 GP sheets (two in each group) was measured successively. A single measurement lasted 15 min and the sampling time was 2 h. Two characteristic curves for the calculation of GP water content from the measured remaining charge were derived for two different positions as shown in Figure 4. The capacitor circuit was discharged through the GP between the indicated measurement positions and the ground. Only one measurement position was connected at a time.

**Figure 5:**
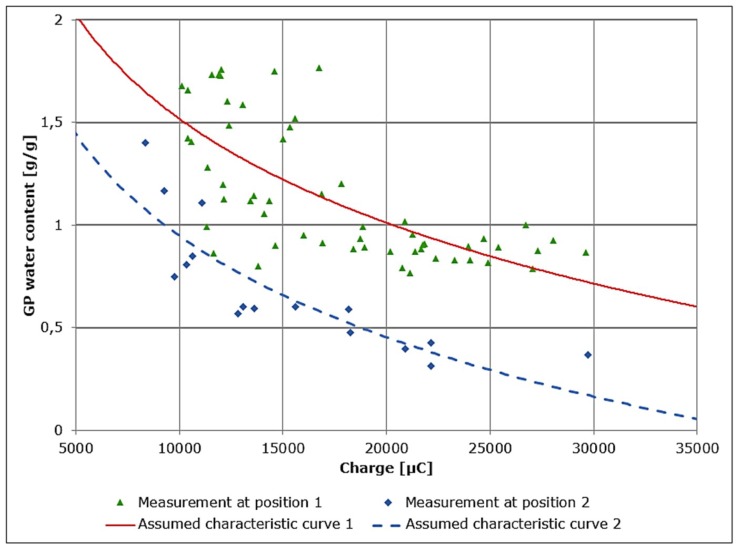
GP water content and remaining charge for the two different measurement positions.

The data were collected by repeated measurements of remaining charge and corresponding gravimetric water contents. The gravimetric water contents were measured using a precision balance. The dedicated measurements and characteristic curves are shown in Figure 5. The assumed characteristic curves were generated by numerical fitting assuming a logarithmic relationship between GP electrical resistance (and, by extension, water content) and remaining charge as suggested by Equation (2) [8].

The evaluation of the measurement accuracy shows a mean deviation of automated measurements from gravimetric measurements of more than ±0.44 g/g for the whole effective range. This initial measurement accuracy, though typical for the implemented circuit, is unfortunately not sufficient enough to be used for the effective control of GP water content. From Figure 5 the practical problem resulting from the use of GP in combination with direct measurements from the automated measurement system can be clearly detected. From a theoretical point of view, the measurements are not accurate or can be assumed as noisy. To overcome this problem using GP, a new (filtered) measurement approach has to be developed. A similar problem is known from studies involving measurement of soil moisture content [10], where filters are also employed as solutions.

### 2.3. Improved Model-Based Measurement System

Uncertainties in a system measurement often limit the performance of controllers. Observers, filters, and especially Kalman filters were developed in the last few decades to improve estimations of real states resulting from noisy measurements and/or noisy processes/systems (systems with randomly varying system parameters). Kalman filters have been applied in Groenendyk et al. [10] and Rosnay et al. [11], combining model-based predictions of soil moisture content with in-situ measurements to generate more reliable moisture content estimates.

**Figure 6:**
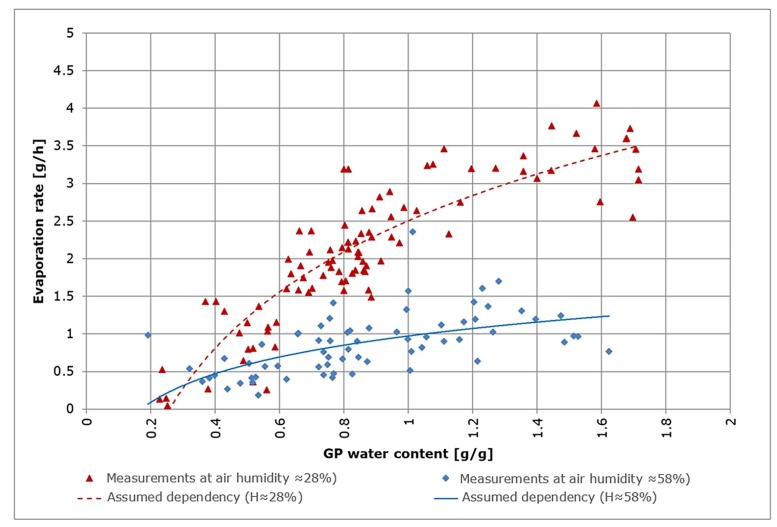
Evaporation and GP water content for two different values of air humidity (28% and 58%).

In this work, for measurement improvement, a specific filtering approach was employed. A model for time behavior of GP water content was developed to generate a prediction, which was combined with actual measurements based on correction factors derived from the mean variation of measurements. The model equation:

(3) $$m_{GP}W_{k}=m_{GP}W_{k-1}+I_{k-1}-E_{GP}T_{0},$$

with,

$m_{GP}$: GP mass;
   $W_{k}$: Current gravimetric water content;
$W_{k-1}$: Gravimetric water content at previous time step;
   $I_{k-1}$: Irrigation amount at previous time step;
   $E_{GP}$: GP evaporation rate, and;
     $T_{0}$: Sampling time;

is based on the assumption that the evaporation $E_{GP}$ depends on the GP water content. Preliminary tests showed a logarithmic dependency between the water content of GP and the rate of evaporation shown in Figure 6. This is consistent with a previously documented relationship between evaporation and soil water depletion [12]. Measurements were carried out gravimetrically using a precision balance. The two curves show the assumed dependencies between evaporation and GP water content. From these results, a strong (and formalizable) dependency on air humidity can be assumed.

To prospect these dependencies on evaporation the Penman equation as expressed in Ostrowski [13] as:

(4) $$E_{pot}=\frac{s}{s+\gamma }\frac{E_{R}^{net}}{L}+\frac{\gamma }{s+\gamma }f\left(v\right)\left(e_{s}-e_{a}\right)$$

is employed, where,

(5) $$e_{a}=\frac{e_{s}H}{100}$$

is assumed with:

$E_{pot}$: Potential evaporation;
      *s*: Slope of saturation vapor pressure curve;
      γ: Psychrometric constant;
   $E_{R}^{net}$: Net irradiance;
      *L*: Latent heat of vaporization;
      $e_{s}$: Saturated vapor pressure;
      $e_{a}$: Vapor pressure of free flowing air;
      *H*: Relative humidity, and;
f(v): Wind velocity function.

The parameters saturation vapor pressure gradient *s*, wind velocity *v*, net irradiance $E_{R}^{net}$, and latent heat equivalent *L* are assumed to be constant. From this the dependency:

(6) $$E_{pot}=C_{1}+C_{2}\left(1-H/100\right),$$

using constant terms $C_{1}$ and $C_{2}$ defining the relation between the relative air humidity *H* and the potential evaporation $E_{pot}$, the evaporation from the germination paper, $E_{GP}$, can be derived. The resulting equation for GP evaporation is:

(7) $$E_{GP}\left(H,W_{k}\right)=\left(c_{1}H+c_{2}\right)\ln\left(m_{GP}W_{k}\right)-c_{3}H+c_{4}.$$

This model is fitted to the measurements using coefficients $c_{1}$ to $c_{4}$ (Figure 6) resulting in:

$c_{1}=$ −0.038292758;
$c_{2}=$ 2.901962527;
$c_{3}=$ −0.096373808, and;
$c_{4}=$ 7.18246835.

The transpiration influence can be neglected since the surface of plants leaves at the growth stage of interest in the studies related to this work (3 to 5 leaves) was significantly smaller than the surface of GP sheets. Measurements of evapotranspiration from plants at a similar growth stage grown under identical environmental conditions in 200 ml PET pots were found to be lower than 1 g/h, which is significantly lower than the evaporation from the GP sheets.

**Figure 7:**
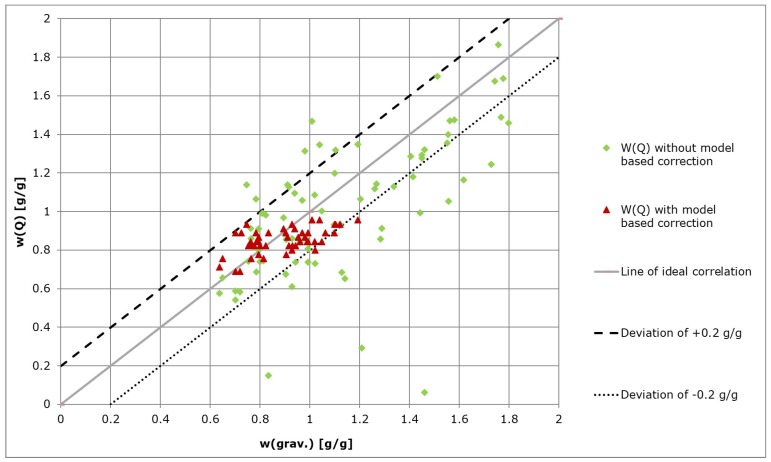
Automated measurements of GP water content W(Q) with and without model-based correction (Kalman filtering).

The automated measurements with model-based correction showed a significantly better correlation to the gravimetrically measured water contents. Implementation of the Kalman filter resulted in the elimination of measurement outliers and consequently a more accurate representation of the system state was obtained as compared to direct measurements taken from the system (Figure 7).

### 2.4. GP Water Content Control (MPC and PI)

**Figure 8:**
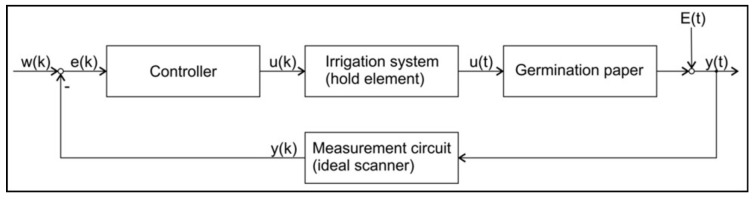
Control loop for the control of GP water content. w(k)—reference variable; e(k)—control deviation; u(k)—calculated irrigation volume; u(t)—irrigation volume; E(t)—evaporation; y(t)—GP water content; y(k)—measured GP water content. (k) denotes a variable in discrete time; (t) denotes a variable in continuous time.

The control loop for GP water content control is shown in Figure 8. The irrigation element was assumed as a zero order hold element. The measurement circuit was assumed as ideal. The evaporation was considered as a disturbance variable acting directly on the output.

The system to be controlled (GP) is modeled as an integral transfer element. Including the hold element of zero order the *z*-transfer function:

(8) $$G_{1}\left(z\right)=\frac{T_{0}}{T_{1}}\frac{1}{1-z}$$

is deduced. From the transfer function as described by Equation (8), two controllers are derived: A model predictive controller (MPC) based on Equations (3) and (7) [14] and a time discrete PI-Controller [15] described as:

(9) $$G_{R,PI}\left(z\right)=\frac{1-0.6z^{-1}}{1-z^{-1}}.$$

The MPC approach was selected because it allowed the quantization of the actuation as well as related limitations. Both controllers were applied to realize two different control tasks, which are:

(i) An individual control of the water content of a single sheet of GP and;
(ii) A group control whose implementation is subsequently described:
  The mean value of the two automated water content measurements from each group under consideration was calculated. Subsequently a single irrigation volume was calculated by the controller. The irrigation volume was applied to all five GP sheets of the group. Therefore, the GP sheets without water content measurement within the group were controlled by an open-loop control.

### 2.5. Testing

A test run was carried out:

(i) To test the accuracy of the water content measurement;
(ii) To validate the functionality of the described control, and;
(iii) To compare the accuracy of the two controller types defined by the control deviations and the integral of squared error.

Test groups A and C were controlled via individual control mode (two GP sheets each). Groups B and D were controlled via group control mode (five GP sheets each). Groups A and B were controlled using the discrete PI-controller while groups C and D were controlled using MPC. The reference variable was set to be 0.89 g/g for five days and afterwards 0.56 g/g for four days for all groups. The ambient conditions were adjusted as stated above. The GP sheets were weighed twice a day using a precision balance. These measurements were taken as reference for the evaluation of accuracy of the automated measurements.

## 3. Results

### 3.1. Preliminary Tests

**Figure 9:**
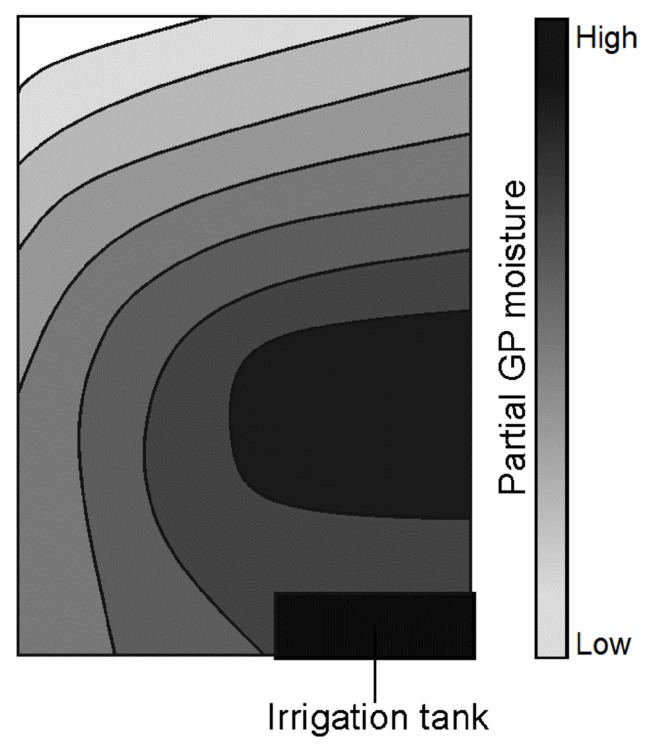
Qualitative distribution of nutrient solution within a GP sheet.

The investigation of distribution of nutrient solution in GP over time shows a heterogeneous distribution pattern dependent on total GP water content, as qualitatively shown in Figure 9. The distribution gradient increased as the GP water content decreased, indicating an acceleration of evaporation with time.

Drying out the GP from saturation and evaluation of the water distribution indicated greater evaporation from the corner samples. This was due to a greater exposure to the environment at the frame boundary. Similarly, the left edge (Figure 9) experienced greater exposure to the atmosphere as a result of facing the interior of the greenhouse. This is assumed to be the reason for the left-right gradient of water distribution obtained. Gravitational effects would also contribute to faster loss of water from the topmost sections.

### 3.2. Accuracy of Water Content Measurements

The accuracy of the automated water content measurements was defined by the maximal difference between the automated measurements and the gravimetrical measurements as shown in Table 2. An accuracy of ±0.2 g/g was assumed to be sufficient for most applications within the experimental setup employed. Therefore the effective range was defined to 1.64 g/g >W> 0.80 g/g for measurement position 1 and to 0.66 g/g >W> 0.45 g/g for measurement position 2. Outside of these ranges, the automatic measurements are not accurate enough to be used for an effective control of GP water content. Variations of 0.2 g/g are large in comparison with a maximum water content of 2.0 g/g. It is assumed that these variations can be minimized by correction using a look-up table and elimination of disturbances within the measurement unit.

### 3.3. Assessment of Water Content Control

**Figure 10:**
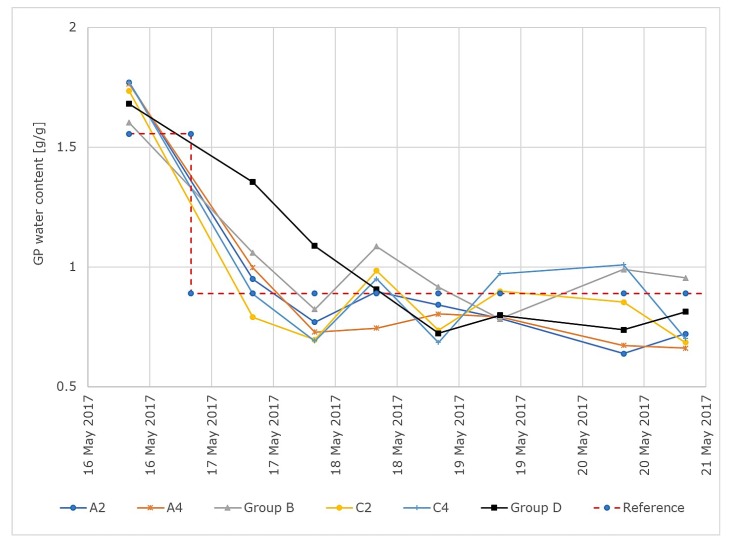
Results of the control test run for a reference variable of 0.89 g/g. GP moisture values over time for all tested GP sheets. Groups A and C were controlled individually and groups B and D were controlled in group control mode.

The gravimetric GP water content over time for all groups and for a reference variable of 0.89 g/g is shown in Figure 10.

The control deviation:

(10) $$e=w-W_{grav}$$

with the reference variable *w* and the gravimetrically measured water content $W_{grav}$ as output variable is evaluated. Additionally, the integral of squared error:

(11) $$A_{quad}=\int_{0}^{\infty }e^{2}dt$$

is evaluated. The maximal control deviations and the integral of squared error of the different groups for w=0.89 g/g are shown in Table 3.

For the plants under group control, the means and standard deviations of the control error within the groups are shown in Table 4. Each group consisted of 5 plants, with 7 measurements for each plant taken into consideration.

## 4. Discussion

The actual GP water content as well as variations in GP water content between different GP sheets over time have not been considered in past studies [1,2,3]. One aim of this work was the development of a method for automated measurement of GP water content allowing the consideration of the above mentioned variables. Another aim was the development of GP water content control using automated measurements. This allows the application of defined water deficits with a variable intensity over time to examine related plant growth. This is not possible with common methods in existing studies [1,2,3,4,5].

The main focus of this work was the development of an automatic measurement of GP water content with an adequately effective range and accuracy. The measurements were realized by a capacitor circuit ultimately measuring the electrical resistance of GP. The measured values were additionally processed by a model-based Kalman filter to improve accuracy.

The effective range of measurement was 1.64 g/g >W> 0.80 g/g (for contact position 1) with a maximal GP water content of 2.0 g/g. This effective range could be adjusted by the reduction of distance between the measurement contacts on the GP sheets. The final accuracy of measurements (including model-based correction) within the effective range was smaller than ±0.2 g/g.

The control sytem performance was tested at two different levels of GP water content. For the experiments, a MPC was used to reduce the control deviations of the implemented control loop. The best control result in the test run was realized in a single group of GP sheets with a maximal control deviation of 0.09 g/g.

The application of MPC resulted in smaller maximal control deviations when compared to a time discrete PI-controller. Based on the integral of squared error, a direct advantage of one of the two controllers over the other cannot be ascertained. However, both controllers induced similar dynamical behavior. The best control result was obtained using a MPC applied in group control mode.

The control output in group control was based on the measurements from only 2 plants per group which were used for the control of the water content of all the plants in the group. From the results, it can be concluded that this method could be used to improve the control of GP water content based on measurements taken from a sample of GP sheets rather than from all individuals.

The quality of water content control strongly depends on measurement accuracy. The improvement of measurement accuracy in future could be achieved by improving the measurement circuit and by the reduction of disturbance values acting on the measurement.

The system allows the application of defined time-varying intensities of water deficit on plants grown on GP. Therefore it can be used to prospect the dependencies between dynamically changing water deficit and plant development. The system for measurement of GP water content can also be applied to test rigs using a pouch-and-wick configuration to identify variations in GP water content, which are not considered in many studies [1,2,3].

Causes of the differences between test groups were not explored for this work. The assumption was that these differences are either caused by differences in GP attributes or by local variations of surrounding conditions like temperature or wind.

## 5. Summary and Conclusions

Plant experiments involving water deficit require specific knowledge about the moisture available to the plant for control of irrigation. In studies using germination paper as a substrate, moisture measurement and precise watering based on the individual water uptake situation is difficult due to extreme measurement noise.

A novel method for improved measurement and suitable control of GP water content was described in this work. The measurement principle was based on the relationship between electrical resistance of GP, determined via capacitance measurements, and the moisture content of the GP. Kalman filtering was applied for improved accuracy. The control of GP water content was implemented using a model predictive controller.

The measurement system allowed for accurate determination of water content within the 0.8 g/g to 1.64 g/g range for GP with maximal water content of 2.0 g/g. The measurement range could be further extended by adjusting the positioning of the electrical contacts during measurements. The model predictive controller allowed the GP water content to be maintained at predetermined levels, with the ability to vary the desired GP water content over time. System measurement accuracy was ±0.22 g/g and control accuracy was ±0.09 g/g.

The introduced system strongly improved the existing technology and helped to improve sensing and actuation within lab procedures. The application of the system would allow continuous tracking of GP moisture content which is crucial in mapping dependencies between water content and plant behavior under water deficit conditions. The system also allows the maintenance of GP at dynamically varying water content values, which would allow the exploration of controlled variable water deficit sequences in plant growth studies.

Further work on the distribution of water on GP during water uptake would be useful in mapping out the actual water availability at different sections of the GP.

## Figures and Tables

**Table 1:** Available GP moisture measurement methods.

Measuring Equipment	Measurement Principle	Results
Multimeter	Direct measurement of electrical resistance	Inconsistent values obtained
Air humidity sensor	Measurement of air humidity in a capsule attached to the GP	Inconsistent values obtained
Capacitor with GP as dielectric	Measurement of capacitance	No dependence on GP water content observed
Capacitor circuit with GP as resistance	Measurement of electrical resistance via capacitor discharge	Dependency on GP water content observed

**Table 2:** The results of the measurement accuracy’s evaluation. The measurement accuracy is defined as the maximal deviations of automated measurements from the manual measurements. The accuracy is evaluated for different ranges of retained GP moisture.

GP Moisture W	Position 1 Accuracy	Position 2 Accuracy
W > 1.67 g/g	±0.27 g/g	>±0.67 g/g
1.64 g/g > W > 1.02 g/g	±0.16 g/g	>±0.67 g/g
1.01 g/g > W > 0.80 g/g	±0.16 g/g	±0.69 g/g
0.79 g/g > W > 0.67 g/g	±0.31 g/g	±0.42 g/g
0.66 g/g > W > 0.45 g/g	>±0.67 g/g	±0.27 g/g

**Table 3:** Maximal absolute control deviations and integrals of squared error for the different test groups.

Group/Control Applied	Max. Absolute Control Deviation	Integral of Squared Error
A2 (PI-Individual)	0.25 g/g	0.474
A4 (PI-Individual)	0.23 g/g	0.650
B (PI-Group)	0.23 g/g	0.453
C2 (MPC-Individual)	0.20 g/g	0.450
C4 (MPC-Individual)	0.20 g/g	0.565
D (MPC- Group)	0.09 g/g	0.329

**Table 4:** Medians and standard deviations of control error for group-controlled plants.

Group	Median of Control Error	Standard Deviation of Control Error
B	0.08 g/g	0.185
D	0.04 g/g	0.147

## Abbreviations

The following abbreviations are used in this manuscript:

CAN	Controller area network
GP	Germination paper
MPC	Model predictive controller
PEG	Polyethylene glycol
PLC	Programmable logic controller
